# Coexistence of perseveration and apathy in the TDP-43^Q331K^ knock-in mouse model of ALS–FTD

**DOI:** 10.1038/s41398-020-01078-9

**Published:** 2020-11-04

**Authors:** Eosu Kim, Matthew A. White, Benjamin U. Phillips, Laura Lopez-Cruz, Hyunjeong Kim, Christopher J. Heath, Jong Eun Lee, Lisa M. Saksida, Jemeen Sreedharan, Timothy J. Bussey

**Affiliations:** 1 Department of Psychiatry, Institute of Behavioral Science in Medicine, Brain Korea 21 Plus Project for Medical Sciences, Yonsei University College of Medicine, Seoul, Republic of Korea; 2 Department of Psychology and MRC/Wellcome Trust Behavioural and Clinical Neuroscience Institute, University of Cambridge, Cambridge, UK; 3 Department of Basic and Clinical Neuroscience, Maurice Wohl Clinical Neuroscience Institute, Institute of Psychiatry, Psychology and Neuroscience, King’s College London, London, UK; 4 Department of Physiology, Development and Neuroscience, University of Cambridge, Cambridge, UK; 5 School of Life, Health and Chemical Sciences, The Open University, Walton Hall, Milton Keynes, UK; 6 Department of Anatomy, Yonsei University College of Medicine, Seoul, Republic of Korea; 7 Molecular Medicine Research Laboratories, Robarts Research Institute & Department of Physiology and Pharmacology, Schulich School of Medicine & Dentistry, Western University, London, ON Canada; 8 The Brain and Mind Institute, Western University, London, ON Canada

**Keywords:** Psychiatric disorders, Learning and memory

## Abstract

Perseveration and apathy are two of the most common behavioural and psychological symptoms of dementia (BPSDs) in amyotrophic lateral sclerosis–frontotemporal dementia (ALS–FTD). Availability of a validated and behaviourally characterised animal model is crucial for translational research into BPSD in the FTD context. We behaviourally evaluated the male TDP-43^Q331K^ mouse, an ALS–FTD model with a human-equivalent mutation (TDP-43^Q331K^) knocked into the endogenous *Tardbp* gene. We utilised a panel of behavioural tasks delivered using the rodent touchscreen apparatus, including progressive ratio (PR), extinction and visual discrimination/reversal learning (VDR) assays to examine motivation, response inhibition and cognitive flexibility, respectively. Relative to WT littermates, TDP-43^Q331K^ mice exhibited increased responding under a PR schedule. While elevated PR responding is typically an indication of increased motivation for reward, a trial-by-trial response rate analysis revealed that TDP-43^Q331K^ mice exhibited decreased maximal response rate and slower response decay rate, suggestive of reduced motivation and a perseverative behavioural phenotype, respectively. In the extinction assay, TDP-43^Q331K^ mice displayed increased omissions during the early phase of each session, consistent with a deficit in activational motivation. Finally, the VDR task revealed cognitive inflexibility, manifesting as stimulus-bound perseveration. Together, our data indicate that male TDP-43^Q331K^ mice exhibit a perseverative phenotype with some evidence of apathy-like behaviour, similar to BPSDs observed in human ALS–FTD patients. The TDP-43^Q331K^ knock-in mouse therefore has features that recommend it as a useful platform to facilitate translational research into behavioural symptoms in the context of ALS–FTD.

## Introduction

Amyotrophic lateral sclerosis (ALS) and frontotemporal dementia (FTD) exist on a clinicopathological spectrum, referred to as ALS–FTD complex or FTD with motor neuron disease^1,2^. While patients diagnosed with ALS primarily present with motor deficits and degeneration of motor neurons, FTD, especially the behavioural variant subtype, is usually associated with cognitive and behavioural symptoms. However, particularly distressing to both patients with FTD and their caregivers are the behavioural and psychological symptoms of dementia (BPSDs), which damage quality of life and accelerate disease progression, leading to earlier institutionalisation^3–6^. Two of the most common BPSDs seen in FTD or ALS–FTD are perseveration (inappropriate repetitive behaviour) and apathy (reduced motivation)^5–9^. Despite the substantial impact of BPSD in FTD, our understanding of BPSD in these patients is still lacking. One of the reasons for this may be a limited availability of translationally appropriate animal models of ALS–FTD, and reliable methodologies with which to study complex behaviours in such models.

Almost all cases of ALS and half of FTD cases are characterised by pathological inclusions of the RNA-binding protein TDP-43^10,11^. The identification of mutations in the gene encoding TDP-43 (TARDBP) in ALS and FTD indicates a mechanistic link between TDP-43 and neurodegeneration^12,13^. To better understand the role that TDP-43 plays in disease, we recently created a novel ALS–FTD mouse model harbouring a human-equivalent point mutation (TDP-43^Q331K^) in the endogenous mouse *Tardbp* gene^12,14,15^. We found that the TDP-43^Q331K^ knock-in mouse (referred to herein as the TDP-43^Q331K^ mouse) exhibits cognitive symptoms typical of FTD such as inattention and memory impairment^14^. The TDP-43^Q331K^ mouse also demonstrates altered splicing of the *Mapt* gene, which encodes the protein tau, which is also closely associated with FTD pathogenesis^16^. This knock-in model may have greater translational value than other existing transgenic models as the observed phenotypes cannot be overexpression artefacts and are therefore more likely to reflect pathogenic changes that occur in human ALS–FTD^14^.

Given the cognitive similarities between the TDP-43^Q331K^ mouse and ALS–FTD, here we evaluated these animals for evidence of abnormal behaviours consistent with human BPSDs, using the touchscreen-operant chamber system, which enables comprehensive and sensitive behavioural measures across multiple domains^17^. Specifically, we report that the male TDP-43^Q331K^ mice exhibit perseveration and evidence of apathy-like behaviour. Our findings highlight the utility of this animal model and the touchscreen-operant system in studying the behavioural symptoms of ALS–FTD.

## Materials and methods

### Animals

**Fig. 1:**
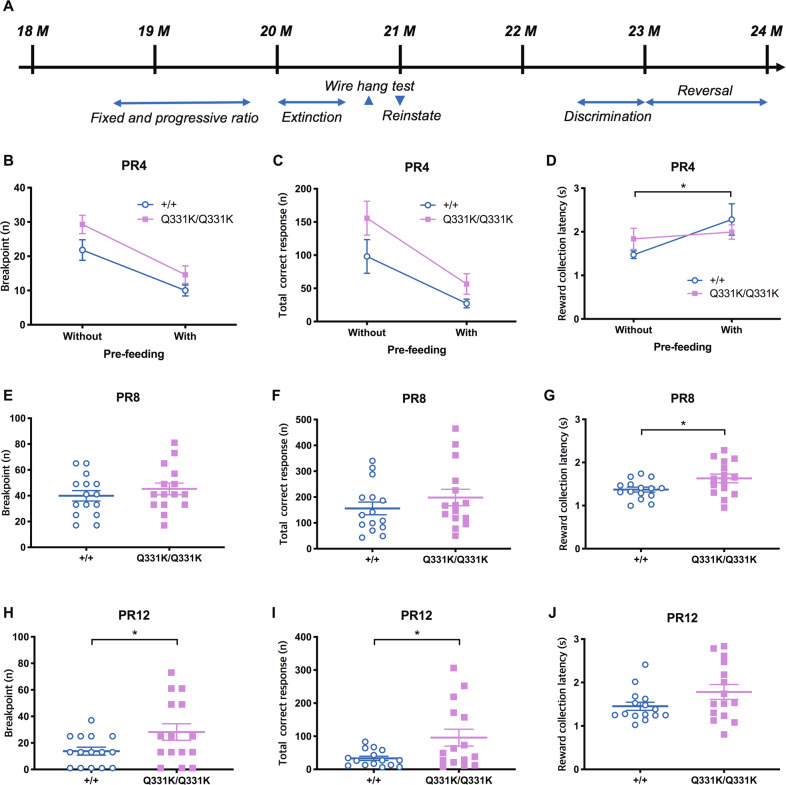
Progressive-ratio (PR) schedules show enhanced responding in TDP-43^Q331K^ mice. **A** Behavioural assessments and the age of animals at which these tasks were conducted. **B**–**J** Motivation levels were evaluated using PR schedules. **B** PR4 breakpoint; main effect of genotype, *F*(1,30) = 4.08, *P* = 0.052; main effect of feeding, *F*(1,27) = 62.81, *P* < 0.0001; genotype × feeding interaction, *F*(1,27) = 0.53, *P* = 0.474. **C** Total number of correct responses; main effect of genotype, *F*(1,30) = 3.69, *P* = 0.064; main effect of feeding, *F*(1,27) = 33.99, *P* < 0.0001; genotype × feeding interaction, *F*(1,27) = 0.84, *P* = 0.368. **D** Reward collection latency; main effect of genotype, *F*(1,30) = 0.02, *P* = 0.881; main effect of feeding, *F*(1,28) = 4.43, *P* = 0.044; genotype × feeding interaction, *F*(1,28) = 1.94, *P* = 0.174. **E** PR8 breakpoint; *P* = 0.388. **F** PR8 total number of correct responses; *P* = 0.306. **G** PR8 reward collection latency; *P* = 0.035. **H** PR12 breakpoint; *P* = 0.047. **I** PR12 total number of correct responses; *P* = 0.025. **J** PR12 reward collection latency; *P* = 0.102. *n* = 15–16 per genotype.

Generation of the TDP-43^Q331K^ knock-in mutant mouse was described previously^14^. A total of 32 aged-matched male mice (16 homozygous mutants, TDP-43^Q331K/Q331K^ and 16 littermate wild types, TDP-43^+/+^) were housed in groups of 2–5 per cage under a 12-h light/dark cycle (lights on at 7:00 pm). Sample size for behavioural testing was based on our historical touchscreen data, which indicates thr required group sizes of 15 animals per genotype to detect an ~20% difference in performance between groups. The order and genotype of animals tested were randomised by one operator before subsequent experimental studies were conducted by a second investigator^14^. All behavioural testing was conducted during the dark phase. Animals were evaluated by a series of behavioural tasks from 8 to 24 months of age (Fig. 1A). Animals were food-restricted so that they were maintained at 85% of free-feeding weight by daily provision of chow pellets (RM3, Special Diet Services, Essex, UK). Drinking water was available ad libitum. All experiments were conducted in accordance with the United Kingdom Animals (Scientific Procedures) Act (1986) and the United Kingdom Animals (Scientific Procedures) Act (1986) Amendment Regulations 2012 and reviewed and approved by the University of Cambridge AWERB.

### Apparatus and reagents

Cognitive testing was performed in Bussey-Saksida touchscreen chambers (Campden Instruments Ltd., Loughborough, UK) as detailed elsewhere^17^. Milkshake (Yazoo^®^, FrieslandCampina UK, Horsham, UK) was used as an appetitive operant reinforcer^18^.

### Fixed and progressive ratios (FR/PR)

The animals involved in this study had previously undergone touchscreen-based cognitive testing including the 5-choice serial reaction time task (5-CSRTT) and FR/PR schedules (at 7 months of age) as reported in our prior publication^14^, thereby enabling a condensed behavioural training programme to be used, with apparatus habituation and Pavlovian pretraining phases omitted. The touchscreen FR and PR schedules have been described previously^19,20^. Briefly, animals were first trained to complete 30 trials in 30-min FR1 sessions and trained to FR3 and FR5. After completion of FR5, animals were evaluated in PR schedule, in which the reward response requirement was increased on a linear + n basis (i.e., in PR4; 1, 5, 9 and 13) upon completion of each trial. The sessions terminated if no response to the screen or magazine entry was detected in 5 min. PR performance was evaluated by breakpoint, which is defined as the number of target responses emitted in the last successfully completed trial of a session.

As we had previously observed hyperphagia in TDP-43^Q331K^ mice^14^, we also assessed performance on the PR4 schedule following milkshake prefeeding to control for potential genotype differences in reinforcer valuation^21^. In prefeeding sessions, animals were allowed 60 min of free access to a bowl of milkshake put in the touchscreen chamber prior to PR testing.

To further characterise PR performance, we also conducted within-session response rate analysis in the first PR session, as previously described^18,21–23^. The total response time (TRT) of each trial was converted to rate (responses per minute) and fitted with the equation, *y* = *a*^(−b*x), using non-linear least-squares regression, in which x indicates the number of trials and y is the response rate. From this model, we obtained predicted values for the peak response rate (*a*) and decay rate (*b*) for individual animals. The peak response rate (*a*) suggests the maximum level of motivation of each animal to obtain reward, while the decay rate (*b*) indicates how this motivation diminishes across subsequent trials. Here we used this analysis on data collected from the PR4 and PR8 schedules as we found that the PR12 schedule resulted in data that could not be reliably fitted to the model equation. All touchscreen testing was conducted blind to genotype.

### Evaluation of food intake

To assess for hyperphagia (a clinical feature of FTD), we measured food intake in two ways. Firstly, we measured the intake of freely available food by placing individual animals in touchscreen chambers with a bowl of strawberry milkshake for 60 min. Secondly, we determined the effect of introducing a low-effort work requirement on intake by assessing animals in a 60-min FR1 session where the maximum number of trials available was uncapped (FR1-uncapped). In both cases, animals were assessed in a familiar environment (the touchscreen chamber to which they had been previously habituated), thereby providing measures of food intake in low-stress conditions. These procedures were performed 60 min before animals routinely received their daily allocation of RM3 diet.

### Extinction task

Animals were first trained to complete 30 FR1 trials in 12.5 min for 5 consecutive days. During the following extinction phase, the same screen stimulus was displayed in the same screen location, but disappeared when touched (response) or after 10 s of presentation (omission). No reinforcement was provided, regardless of response or omission. Each extinction session consisted of 30 trials with a 4.5-s ITI. The extinction task was performed until trial omission reached at least 77% in two consecutive sessions. Ten days after the last extinction session, animals underwent a 12.5-min/30-trial reinstatement session, in which only the first three responses were rewarded^17^.

### Rolling wire hang test

Neuromuscluar strength was evaluated by the rolling wire hang test described elsewhere^24^. Animals first underwent 5-min habituation and were tested on a separate day. Testing was limited to 5 min, and the latency to fall off was recorded. All testing was conducted blind to genotype.

### Visual discrimination and reversal (VDR) task

#### Discrimination acquisition

Detailed procedures of touchscreen visual discrimination are described elsewhere^17,25^. Discrimination-acquisition criterion was ≥80% correct response for 2 consecutive days. Two mice (one from each genotype) which had not reached this criterion in 20 sessions were excluded.

#### Reversal

After discrimination acquisition, animals received two further discrimination sessions to reinforce reward contingencies and ensure stable baseline performance. On the following day, the correct and incorrect contingencies were reversed, and all mice received reversal sessions under the new contingencies until the last (slowest to reverse) mouse reached ≥50% correct response for two consecutive sessions. In contrast to discrimination acquisition, we did not include correction trial in reversal, since the main purpose of this assessment phase was to examine perseveration rather than the learning of a new association, with the prior reported to dominate the reversal phase until performance exceeds 50% correct and at which point new learning begins^25,26^.

### Statistical analysis

Statistical analyses were conducted using R version 3.3.0 (https://www.r-project.org) and Prism version 8 (GraphPad Software, Inc., San Diego, CA, USA). Normality of data was examined by D’Agostino–Pearson omnibus test. Between-group differences were evaluated using *t* test, if needed for different group variance, with Welch’s correction or Mann–Whitney *U* test in cases where data were not normally distributed. Repeated-measures data were analysed by mixed-effects models to identify the main effects of group or session and group-by-session interactions. Data are presented as mean ± standard error (SEM). Significance was set at *α* < 0.05.

## Results

### TDP-43^Q331K^ mice show increased responding under PR schedules

To determine if TDP-43^Q331K^ mice demonstrate reduced motivation (apathy), we used touchscreen FR/PR schedules. Since TDP-43^Q331K^ mice in a free-feeding state showed hyperphagia^14^, we conducted PR4 with and without prefeeding to control for potential genotype differences in appetite or reinforcer valuation. Neither a main effect of genotype or genotype × feeding condition interaction was identified (Fig. 1B, C). As expected, prefeeding was associated with an increased reward collection latency (Fig. 1D), but was not genotype dependent. A trend towards a higher breakpoint in TDP-43^Q331K^ mice was detected under the PR4 schedule.

We then transitioned the mice to systematically more demanding PR schedules (PR8 and PR12) to determine if a greater rate of work-requirement increase would more fully unmask a genotype difference. In PR8, no main effect of genotype was detected on breakpoint or total touches (Fig. 1E, F), but reward collection latency was significantly increased in the TDP-43^Q331K^ group (Fig. 1G).

Unexpectedly, under the PR12 schedule, the TDP-43^Q331K^ mice showed a significantly higher breakpoint (Fig. 1H) and a higher number of total correct touches (Fig. 1I) with a similar reward collection latency relative to wild-type (WT) littermates (Fig. 1J). Blank touches were similar between genotypes across PR schedules (Supplementary Fig. 1A–C), suggesting equivalent target specificity and engagement.

Higher PR breakpoint is canonically associated with increased motivation for reward, although the inconsistent genotype-dependent effects across the different PR schedules observed here suggested a more complex phenotypic presentation. Given the frontal lobe dysfunction, previously characterised in the TDP-43^Q331K^ animals^14^, we speculated that the abnormally enhanced responding we observed under the very strenuous PR12 schedule might be a manifestation of a tendency for these animals to exhibit behavioural perseveration^27^, effectively causing them to over-respond in the PR schedule relative to WT, even though their motivation for reward is not significantly different. Indeed, the probability of the TDP-43^Q331K^ animals exhibiting increased motivation for reward is further reduced by the comparable or higher reward collection latencies (Fig. 1D, G, J) and slower FR acquisition we observed in these animals relative to WT (Supplementary Fig. 1d, e).

### TDP-43^Q331K^ mice show decreased PR response rates

**Fig. 2:**
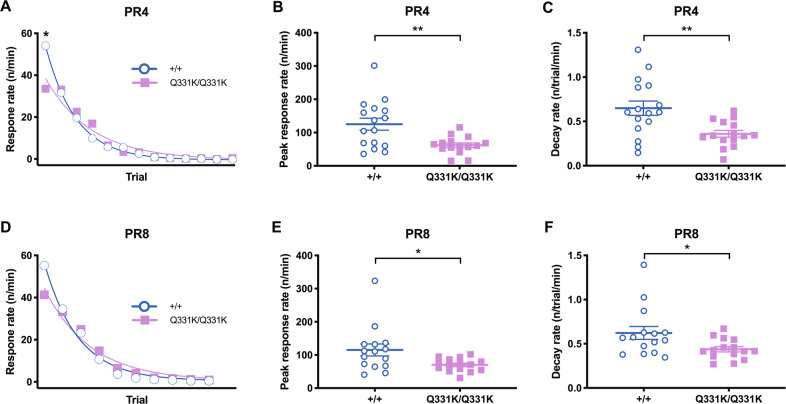
Within-session response rate analyses of PR suggest reduced motivation in TDP-43^Q331K^ mice. Response rate was analysed to evaluate motivation changes within PR sessions. **A** Response rate across trials throughout a session of PR4 (without prefeeding). Main effect of genotype, *F*(1,29) = 0.15, *P* = 0.704; main effect of trial, *F*(12,348) = 74.56, *P* < 0.0001; genotype × trial interaction, *F*(12,348) = 3.66, *P* < 0.0001; simple main effect of group at the first trial, *P* = 0.011. **B** Peak response rate in a PR4 session. *P* = 0.003. **C** Response decay rate in a PR4 session. *P* = 0.004. **D** Response rate across trials throughout a session of PR8. Main effect of genotype, *F*(1,28) = 0.01, *P* = 0.927; main effect of trial, *F*(9,252) = 120.40, *P* < 0.0001; genotype × trial interaction, *F*(9,252) = 2.68, *P* = 0.006; simple main effect of group at the first trial, *P* = 0.062. **E** Peak response rate in a PR8 session. *P* = 0.023. **F** Response decay rate in a PR8 session. *P* = 0.026. *n* = 15–16 per genotype.

To determine if the apparently increased PR responding in the mutant mice was due to perseveration or hypermotivation, we further analysed within-session response rates, as this can reflect changes in animals’ motivation throughout a PR session^18,22^. PR4 response rate showed a significant genotype × trial interaction, with a significant genotype difference in the initial phase where relatively low levels of responding are required (Fig. 2A). Consistent with this, the maximal response rate was significantly lower (Fig. 2B) in TDP-43^Q331K^ mice, indicating a deficit in activational motivation. Response decay rates across trials were also slower in the mutant mice (Fig. 2C). Analysis of the PR8 session also revealed a lower rate of responding at the outset of the session in TDP-43^Q331K^ mice (Fig. 2D–F).

The delayed reduction in response rate in the TDP-43^Q331K^ mice could be indicative of perseveration, in that it could explain how animals with an initially lower level of motivation would persist in continuing to emit PR responses such that they ultimately engage with the task for longer than WT, achieve a higher breakpoint and appear to be equally or even more highly motivated. This effect would be magnified in a strenuous schedule such as PR12, where the frequency of reinforcement is lower, such that WT would be more likely to reach the breakpoint earlier than the perseverating TDP-43^Q331K^ group. Taken together, this within-session rate analysis supports the presence of an apathy-like state in the TDP-43^Q331K^ mice, which would have otherwise been masked by their comparable (in PR4 and PR8) or higher (in PR12) breakpoints.

### Motor function of TDP-43^Q331K^ mice

Motor deficits might have caused the genotype difference in the response rate in PR (Fig. 2). Thus, we analysed beam-break rate in PR sessions to examine locomotor behaviour and also conducted the rolling wire hang test^24^ to examine muscle strength. We found no genotype differences in beam-break rate with only an exception in PR8 (Supplementary Fig. 2a–d). There was no genotype difference in latency to fall and body weight in the wire hang test (Supplementary Fig. 2e, f). Together, these data argue against contribution of motor deficits to PR performance in the mutant mice.

### TDP-43^Q331K^ mice showed no hyperphagia

**Fig. 3:**
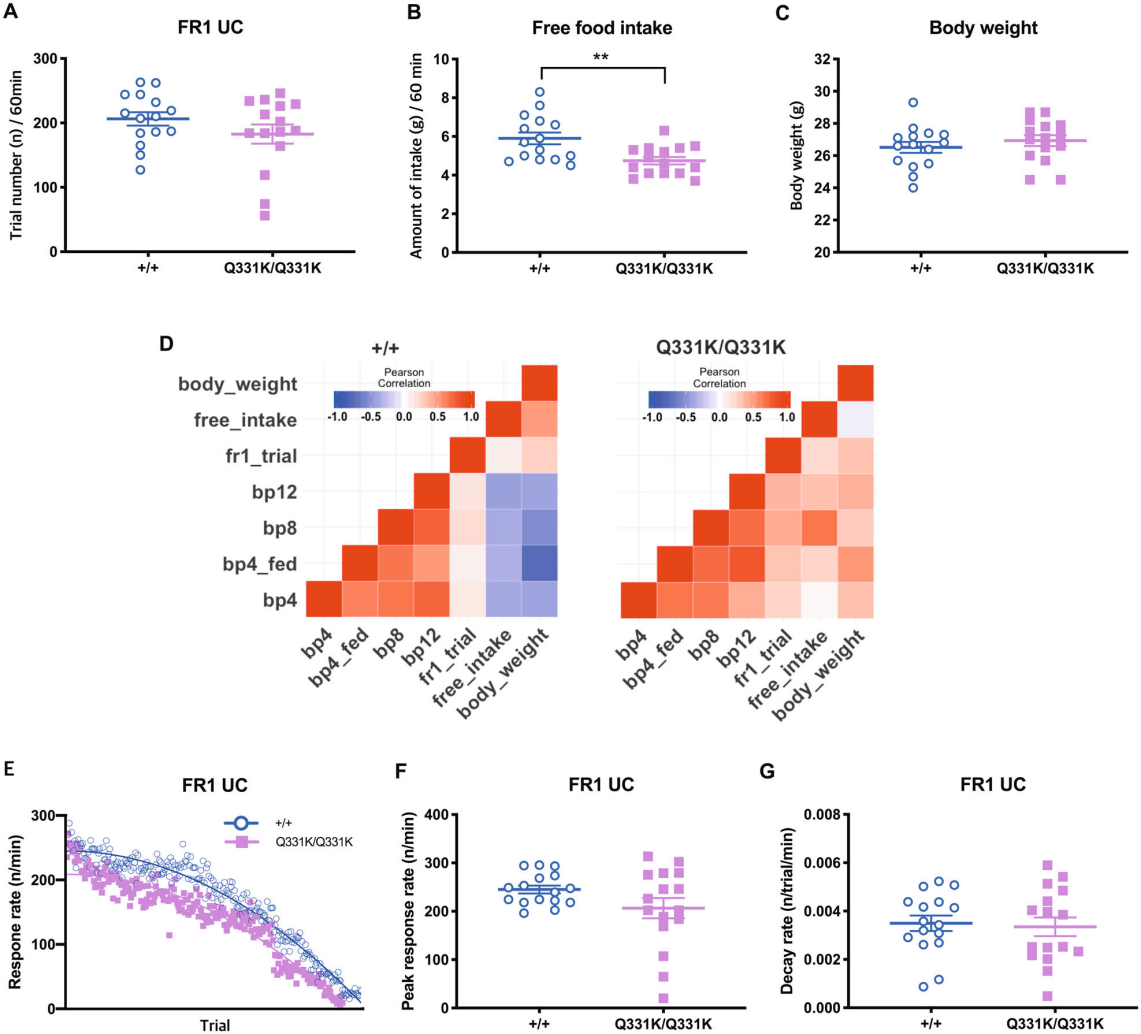
Eating behaviour and its relationship to PR responding. Food intake was measured to rule out hyperphagia in the mutant mice. **A** The total number of rewards earned in an uncapped fixed-ratio1 (FR1-UC) setting over 60 min. *P* = 0.206. **B** Amount of intake with free access to milkshake over 60 min. *P* = 0.003. **C** Body weights. *P* = 0.391. **D** Heatmaps showing the relationship (Pearson’s *r*) between progressive-ratio (PR) breakpoint and food intake and body weight. **E** Changes in response rate through the session of FR1-UC. **F** Peak response rate in FR1-UC. *P* = 0.104. **G** Decay rate in FR1-UC. *P* = 0.774. *n* = 15–16 per genotype. Free_intake amount of milkshake in 60 min, fr1_trial the FR1 trial number, bp breakpoint, bp4_fed breakpoint in PR4 with prefeeding.

We also measured food intake to rule out hyperphagia, which might also have caused increased PR responding in the mutant mice. No effect of genotype was detected in performance on the non-trial-limited FR1 session (Fig. 3A). We then measured food consumption during 60 min of free access to a bowl of milkshake. Unexpectedly, TDP-43^Q331K^ mice consumed less milkshake (Fig. 3B) than WT with comparable initial body weights (Fig. 3C).

These results rule out hyperphagia in the TDP-43^Q331K^ mice. Rather, given that all animals had been food-restricted, and that sweetened strawberry milkshake is highly palatable to mice^19^, reduced free intake may indicate anhedonia in the mutant mice. Based on this speculation, we explored relationships between PR responding, food intake and body weights (Fig. 3D). These relationships were obviously dissociable between the two groups (Fig. 3D). This may suggest a genotype difference in how eating behaviour affects PR responding: in the mutant mice, an overall positive relationship was identified between breakpoints and free milkshake intake (Fig. 3D), supporting the possibility that reduced intake of the free-access milkshake (Fig. 3B) indicates anhedonia in the mutant mice.

To further rule out the possibility that lower appetite or earlier satiation might cause reduced intake of the free-access milkshake in the mutant mice (Fig. 3B), we analysed the response rate in the FR1-uncapped session, by which we can observe temporal changes in satiety^18^. Overall, no significant genotype differences were noted in the response rate change (Fig. 3E), the peak response rate (Fig. 3F) that may reflect the levels of hunger at the initial stage of a session and the decay rate (Fig. 3G), which shows mean satiation rate through the session. Thus, it is unlikely that altered appetite contributed to reduced milkshake intake in the mutant mice.

### TDP-43^Q331K^ mice exhibit increased omissions during early extinction trials

**Fig. 4:**
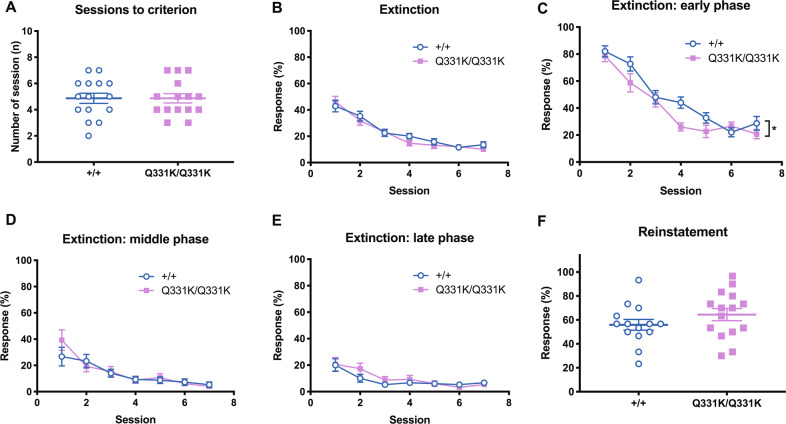
Increased omissions in TDP-43^Q331K^ mice in the early phase of each extinction session. Extinction was tested to assess instrumental responding in the absence of reward. **A** The number of sessions required to reach criterion (>77% omissions in two consecutive sessions) *P* > 0.999. **B** Response percentage across extinction sessions. Main effect of genotype, *F*(1,28) = 0.347, *P* = 0.560; main effect of session, *F*(6,168) = 63.39, *P* < 0.0001; genotype × session interaction, *F*(6,168) = 0.98, *P* = 0.442. **C**, **D** Response percentage in the early (**C**; main effect of genotype, *F*(1,28) = 4.53, *P* = 0.042; main effect of session, *F*(6,168) = 56.70, *P* < 0.0001; genotype × session interaction, *F*(6,168) = 1.72, *P* = 0.117), middle (**D**, n.s. main effect of genotype and genotype × session interaction) and late (**E**, n.s. main effect of genotype and genotype × session interaction) phase of each session. **F** Response percentage in a reinstatement session conducted 10 days after the last extinction session to confirm the degree of extinction learning. Only the first three trials provided reward. *P* = 0.225. *n* = 14–15 per genotype.

As TDP-43^Q331K^ mice showed enhanced responding in PR12 in which reinforcement frequency is very low, we also assessed them in the extinction task under conditions of no reward^28^ to determine if this could interfere with behaviour. Despite our prediction that the extinction task would reveal perseveration in the mutant mice, there was no main effect of genotype on overall extinction performance, as indicated by the comparable number of sessions to criterion (Fig. 4A) and percentage of responses emitted across sessions (Fig. 4B).

We further examined extinction performance by segregating each session (consisting of 30 trials) into early, middle and late phases (10 trials per phase; Fig. 4C, D and E, respectively), since TDP-43^Q331K^ mice showed decreased response rates in the initial phase of each PR session (see Fig. 2) and reduced response in FR training (Supplementary Fig. 1d, e). As expected, TDP-43^Q331K^ animals responded less (exhibited a higher number of omissions) in the early phase (Fig. 4C).

Given the normal overall extinction learning performance observed (Fig. 4A, B), higher omissions restricted to the early phase of a session (Fig. 4C) may suggest reduced task engagement or slow action initiation linked to diminished motivation^29^. The extinction reinstatement session revealed no main effect of genotype on performance (Fig. 4F), suggesting equivalent levels of task acquisition across the genotypes^30^.

### TDP-43^Q331K^ mice show impaired visual discrimination acquisition

**Fig. 5:**
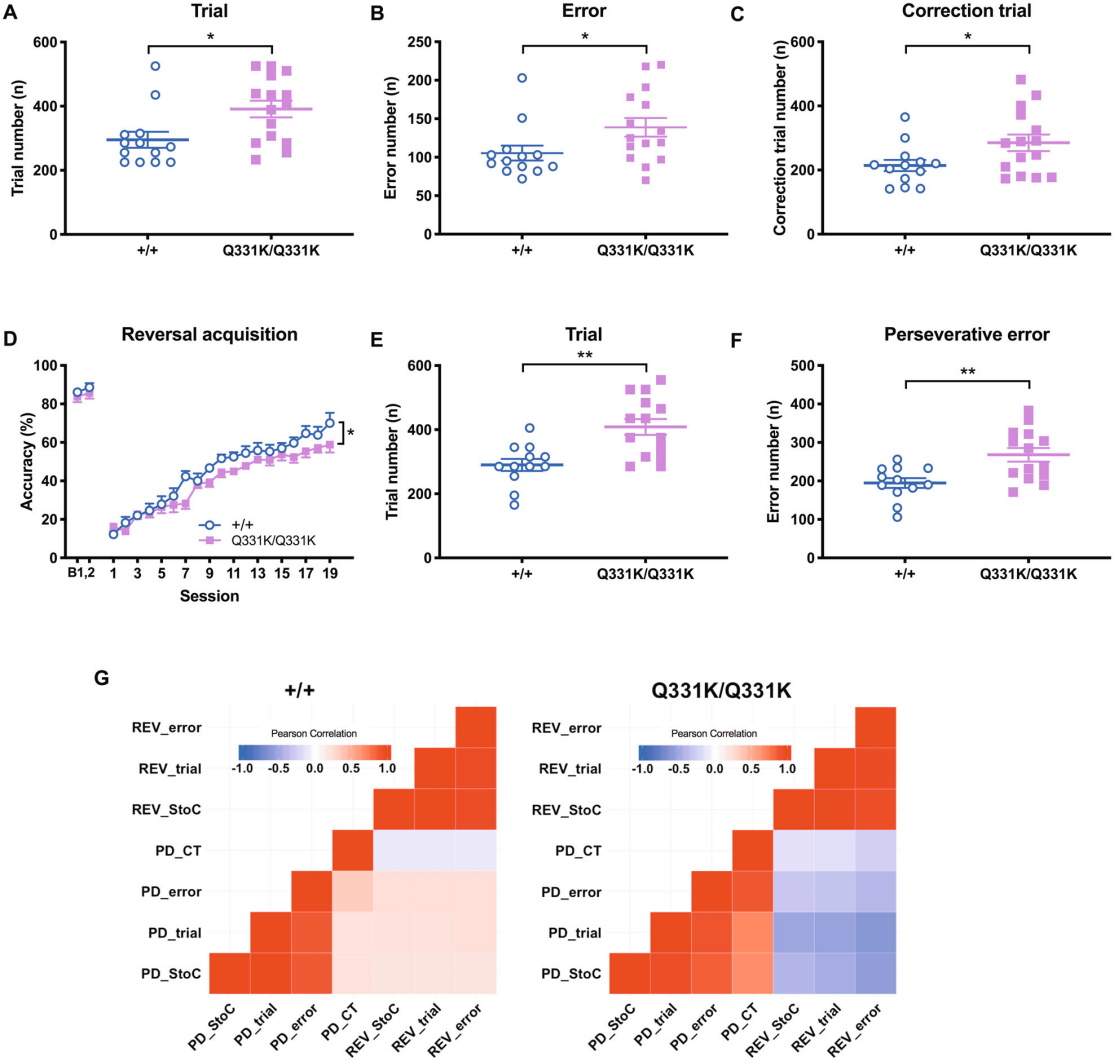
Perseveration in TDP-43^Q331K^ mice revealed by a reversal learning task. Visual discrimination and reversal learning were conducted to assess perseveration. **A** The number of trials required to reach the visual discrimination criterion (>80% accuracy in two consecutive sessions), *P* = 0.012. **B** The number of errors made prior to reaching the discrimination criterion. *P* = 0.028. **C** The number of correction trials required to reach the discrimination criterion. *P* = 0.037. **A**–**C** *n* = 15 TDP-43^Q331K/Q331K^ and 13 TDP-43^+/+^ mice. **D** Baseline performance on two visual discrimination sessions (B1, 2) followed by reversal learning acquisition. Main effect of genotype, *F*(1,24) = 4.81, *P* = 0.038; main effect of session, *F*(18,432) = 75.45, *P* < 0.0001; genotype × session interaction, *F*(6,168) = 1.34, *P* = 0.160. **E** The number of trials required to reach the reversal criterion (>50% accuracy in two consecutive sessions). *P* = 0.001. **F** The number of perseverative errors made prior to reaching the reversal criterion. *P* = 0.003. **G** Heatmaps indicating the relationship of performance between pairwise visual discrimination (PD) and reversal (REV) learning. Trial numbers of PD (PD_trial) were not correlated with numbers of REV trial (Pearson’s *r* = 0.232, *P* = 0.468) or perseverative error (REV_error; *r* = 0.237, *P* = 0.458) in wild types (+/+) but inversely correlated with reversal performances in the mutant mice (for trial numbers, *r* = −0.540, *P* = 0.046; for perseverative errors, *r* = −0.607, *P* = 0.021). StoC number of sessions to criterion, CT number of correction trials. **D**–**G** *n* = 14 TDP-43^Q331K^ and 12 TDP-43^+/+^ mice.

To further explore the possibility of perseveration in TDP-43^Q331K^ mice, we assessed them in the VDR task. TDP-43^Q331K^ mice were slower than WT littermates in acquisition, as indicated by the higher numbers of sessions (Supplementary Fig. 3a), trials, errors and correction trials (Fig. 5A–C) required to reach criterion. S+ (correct) and S– (incorrect) response latencies were also higher in TDP-43^Q331K^ animals than WT (Supplementary Fig. 3b, c), again suggestive of lower motivation. However, motivation to obtain reward did not differ between genotypes as indicated by comparable reward collection latencies (Supplementary Fig. 3d). Also, a genotype difference in locomotor function was not indicated by beam-break rates or time spent in the food tray for reward consuming (Supplementary Fig. 3e–g).

### TDP-43^Q331K^ mice show perseveration during reversal

Following two baseline sessions of the discrimination-acquisition task, which indicated comparable performance between genotypes (Fig. 5D), daily reversal sessions with switched stimulus-reward contingencies began and continued until all mice reached the performance criterion (50% accuracy in two consecutive sessions).

TDP-43^Q331K^ mice were significantly impaired on reversal learning: they were slower in acquisition (Supplementary Fig. 4a and Fig. 5d), required more trials (Fig. 5E) and made more perseverative errors (Fig. 5F) than WT littermates to reach the performance criterion. Genotypes did not differ in either response latency (Supplementary Fig. 4b, c) or reward collection latency (Supplementary Fig. 4d). Also, beam-break rates and time spent to consume reward (Supplementary Fig. 4e–g) showed no genotype differences, ruling out confounding effects of locomotor deficits in mutant mice. Thus, these results indicate cognitive inflexibility in the TDP-43^Q331K^ mice. Specifically, this impaired cognitive flexibility can be attributed to perseveration rather than difficulty in learning a new stimulus-reward association since perseveration dominates reversal performance when accuracy is below 50%^25^. In addition, to further rule out the possibility that deficits in reversal learning in the mutant mice might be only a reflection of their generalised learning difficulty as observed in the visual discrimination task, we examined the relationship between performance on visual discrimination and reversal learning. In contrast to the case of wild-type mice, visual discrimination performance was inversely correlated with reversal performances in the mutant mice (Fig. 5G), supporting that reversal deficits observed in these mice indicate perseveration rather than generalised learning difficulty.

## Discussion

The central finding of this study is that the TDP-43^Q331K^ mouse, a new model of ALS–FTD, exhibits a perseverative phenotype that may mask apathy assessed by PR schedules. These findings are reflective of the two most common BPSDs, perseveration and apathy, in ALS–FTD^5–9^, suggesting that this model may be useful for studying not only the cognitive impairments^14^, but also the behavioural symptoms that have been identified as particularly problematic aspects of this disease.

The TDP-43^Q331K^ mouse may provide an example of a strain, in which increased behavioural responding due to perseveration effectively causes them to ‘over-respond’ in the PR assessment and therefore may mask a motivational deficit. In fact, both perseveration and apathy, while seemingly opposing in nature (an increase vs. a decrease in behavioural output), are highly coincident in FTD, with similar neural correlates^31,32^. It has been found that patients with FTD who display apathy may also show increased reward-seeking behaviour. This increased pursuit of reward may look like increased motivation. However, it is sometimes difficult to determine whether such ‘over-responding’ is due to excessive drive for reward, or to insensitivity to negative feedback. Several clinical studies with bvFTD have suggested that lack of sensitivity to negative consequences is central to abnormal reward processing in FTD^33^. Thus, insensitivity to negative feedback (absence of reward) in the mutant mice might contribute to continuous perseverative responding in the high -ratio schedules (where reinforcement frequency is low) and also to the ‘stimulus-bound perseveration’ in the reversal task^34^. Consistent with this idea, our previous study with mGluR5-deficient mice also showed a possible link between perseveration and increased PR responding, and insensitivity to the absence of reward was attributable to this phenotype^27^.

Of note, a previous study has found that increased reward-seeking behaviour in patients with bvFTD was actually associated with their severity of apathy^35^. This finding may suggest that apathy and abnormally increased reward-seeking behaviour, although seemingly opposite to each other, may stem from a common pathology, such as disturbance in the salience network^36^. In other words, reduced function in this network may cause blunted salience sensitivity not only to negative consequence (absence of reward) but also to newly presented stimulus so that both perseveration and initiation apathy can co-occur. Consistent with this idea, reduced activity in the salience network has been identified in bvFTD (but not in Alzheimer’s disease)^37^. This result may account for the underlying mechanism of abnormal reward processing seen in FTD, as the salience network includes structures known to be involved in reward. Moreover, reduced activity in the salience network has also been associated with the severity of apathy in FTD or late-life depression^38,39^.

Interestingly, our TDP-43^Q331K^ mice displayed reduced responses especially in the initial phase of several tasks: slower acquisition in initial FR1 training (Supplementary Fig. 1), slower initial and peak response rates during a PR session (Fig. 2) and higher omissions in the initial epoch of each extinction session (Fig. 4C). Thus, we also examined response speed in the first trial of the first VDR session and consistently observed slower initiation in mutant mice (Supplementary Fig. 5).

These data may be interpreted in terms of initiation apathy^40,41^. According to a ‘model of goal-directed behaviour’, apathy can be differentiated into three components: initiation, planning and motivation. The Philadelphia Apathy Computerised Task (PACT), which has been developed to measure these three components of apathy in humans, revealed that patients with FTD show a significant impairment in initiation as well as in other components^41,42^. Interestingly, the way to detect initiation apathy using the PACT is very similar to how we interpret slowed response rate in PR sessions as initiation apathy: the PACT criteria for initiation apathy require slowed response time in the first trial of a simple reaction task without generalised slowness^41^. According to this criterion, more generalised slowness in response speed in complex task conditions could not be interpreted as initiation apathy, but may indicate motor deficits^41^.

In a similar vein, detailed consideration of the behavioural profile of the mice indicates an explanation in terms of initiation apathy, and not of motor slowness in general. First, as shown in Fig. 2A and D, the response rate of the mutant mice was slower only at the initial part of the session, which is highly consistent with initiation apathy detected in the computerised test for human patients (PACT)^41^. After this initial slowness, the mean response rate of mutant mice was, although not statistically significant, higher than that of wild-type mice at several trials. This rules out an interpretation in terms of a general, pervasive slowing of behavioural output. If the mutant mice had been generally slower (e.g. due to ALS-related motor deficits), one would expect such motor deficits to be observed throughout the session across the trials, and also in other measures or tasks such as response and/or reward latencies, beam-break rate, rotarod and wire hang test. In addition, the total running time of each session was not significantly different between genotypes (*P* = 0.648 for PR4; *P* = 0.862 for PR8; *P* = 0.282 for PR12), arguing against slower PR performance in mutant mice. This analysis shows that initiation apathy explains the full behavioural profile, and a general motor slowness does not. Of note, it has been shown that initiation apathy significantly increasing in patients with ALS was independent of motor dysfunction^8^.

Altered appetite could be another confounding factor in our study; both abnormally increased and decreased appetite might cause perseverative responding and reduced task initiation, respectively. In the previous study^14^, we observed significant hyperphagia in the mutant mice, a symptom that is also observed in patients with FTD or ALS–FTD^7,43^. However, we found no evidence of hyperphagia in the present study; instead, we observed reduced intake of highly palatable milkshake (Fig. 3B), which if anything may indicate anhedonia in the mutant mice. Moreover, the trial-by-trial analysis of response rate in the uncapped FR1 session (Fig. 3e–g) did not support the possibility of alterations in appetite or satiation rate in the mutant mice.

Of note, there was a discrepancy regarding eating behaviour between our previous^14^ and current studies. This is likely due to differences in the availability of food across the two experiments: hyperphagia was observed in the TDP-43^Q331K^ mice only when food was always available^14^. Importantly, it has been reported that human FTD patients show hyperphagia even without overtly increased appetite^43,44^. Based on this, we speculate that hyperphagia leading to weight gain of the mutant mice in our previous study^14^ was also mediated by perseverative (compulsive) eating rather than increased appetite. Even mild perseveration could cumulatively increase total intake of food and body weight over a sustained period of constant food availability. Consistent with this interpretation, a positive relationship between body weight and PR breakpoint (especially in the prefeeding condition) was observed in the mutant mice, whereas a negative relationship was observed in the WT mice (Fig. 3D). Importantly, these findings also suggest that the relationships between hedonia, motivation and appetitive drive might be disconnected in the mutant mice. Given the findings of dissociations between breakpoint and initial response speed in PR sessions (Fig. 2), such disconnection may be attributed to the perseverative phenotype in these mice. This implicates that care should be taken in interpretation of PR breakpoint data, which could be dissociated with motivation factors in genetically modified disease models, especially in those with the frontal dysfunction^27^.

Assessment of reversal learning further suggested a perseveration-related phenotype in the mutant mice, given that the genotype effect was expressed when reversal performance was below 50% accuracy^25^. This phase of reversal is considered the period when perseveration dominates behaviour. When reversal accuracy exceeds 50%, new learning is considered to control behaviour, and the learning curves for the TDP-43^Q331K/Q331K^ and WT animals were parallel in this epoch, suggesting intact learning of the new association. Most importantly, performance between visual discrimination and reversal learning was inversely correlated in the mutant mice (Fig. 5G), arguing against the possibility that reversal deficits were due to generalised learning deficits rather than perseveration.

This perseverative phenotype in reversal learning is consistent with frontal-specific cognitive deficits observed in human FTD patients^45^ and also with evidence of frontal/executive dysfunction in the TDP-43^Q331K^ mice: inattention was indicated by increased omission errors in the 5-CSRTT at 6 months of age^14^. This was one of the earliest behavioural abnormalities we identified in these mice, together with marble-burying deficits at 5 months of age. At this age, a reduced number of frontal parvalbumin-positive (PV+) interneurons was identified^14^. Interestingly, it has been reported that a deficiency in PV+ interneurons is associated with both high omission errors in 5-CSRTT^46^ and reversal deficits^47^. Therefore, this suggests a common pathological mechanism underlying both inattention and perseveration in our model. However, since we had found no abnormal FR/PR responding at 7 months of age in the mutant mice^14^, the current findings suggest that perseveration is a relatively late-stage phenotype during the progression of the disease, preceded by inattention and memory deficits.

It should be noted that our interpretation of initiation apathy and perseveration came from different protocols of PR schedule: the former from the rate analysis of PR4 and PR8, and the latter from breakpoint of PR12 (we were unable to fit the PR12 data to a model for the rate analysis). That this conclusion could be reached from two different conditions, PR8 and PR4, gives us confidence in our interpretation. Data from PR12 and reversal learning helped explain why breakpoints were comparable between genotypes in PR4 and PR8, despite the mutant mice exhibiting initiation apathy: if the initial lower response rate was the only reason for the reduced decay rate in the mutant mice, their breakpoints would have been lower than those of WT.

In this regard, our analysis illustrates the advantages of an approach to behavioural phenotyping in which animals are tested on a battery of tasks, in which parameters are varied to load on a construct of interest, and in which each task has its own internal controls, but in which in addition, tasks complement each other and can act as controls for each other. In this way, the most likely interpretation of behaviours can be arrived at by taking into consideration the complete behavioural profile of an animal (for another example, see our recent study of mGluR5 KO mice^27^, which similarly showed perseveration and abnormally high breakpoint in PR): characterising the coexistence of perseveration and apathy in this strain would not have been possible with a single-domain behaviour assay or without refined data analyses such as the PR rate analysis and the temporal segregation analysis of the extinction data. Combined with the high translational potential of the touchscreen system^48,49^, this mouse model will be highly valuable in developing a deeper understanding of the pathophysiology of BPSD in the ALS–FTD context.

This study has several limitations. Firstly, we only used male animals to avoid any behavioural consequences potentially resulting from the common occupancy of the operant chambers and housing rooms by both sexes. This limits generalisation of our findings. Secondly, we did not directly examine depressive-like symptoms such as anhedonia and whether it may or may not correlate with the apathy-like behaviour. Depression is also a very common symptom in neurodegenerative diseases. However, apathy and anhedonia involve the two different systems of motivation, which are complementary yet dissociable^50^. Likewise, it has been shown that apathy exists independent of clinical depression in patients with ALS^9^. Thus, a further study is needed to identify differential manifestation of depressive-like behaviour and apathy in our model. For instance, exploring differential effects of serotonergic versus dopaminergic drugs would help further characterise motivation-related phenotype of this model. Thirdly, although perseveration and apathy are BPSDs most commonly found in human patients with FTD or ALS, it remains to be elucidated whether behavioural findings in this study truly indicate human-equivalent BPSDs or simply endophenotypes of this model animal. Finally, we did not examine region-specific neurodegeneration that may correlate with our behavioural findings. Regional brain volume changes in these mice should be the subject of a future study with a particular interest in the orbitofrontal and anterior cingulate cortex and striatum, all of which have been associated with perseveration and (initiation) apathy^9,25,40,42^.

In conclusion, we report that a recently generated TDP-43 knock-in ALS–FTD mouse model exhibits evidence of perseveration and apathy, the most commonly observed behavioural symptoms in ALS–FTD patients. This study suggests the translational utility of this animal model and highlights the value of a multi-domain behavioural approach in studies of BPSD in neurodegenerative disorders.

## Supplementary information

Supplementary Figures
