# Supplementary material for: Coexistence of perseveration and apathy in the TDP-43Q331K knock-in mouse model of ALS–FTD
Source: Transl Psychiatry. 2020 Nov 4;10:377. doi: 10.1038/s41398-020-01078-9 (PMC7643138; doi:10.1038/s41398-020-01078-9)
Supplement: Supplementary file 1 — Supplementary Figures [file 41398_2020_1078_MOESM1_ESM.docx]

***Supplementary materials for***

**Coexistence of perseveration and apathy in the TDP-43^Q331K^ knock-in mouse model of ALS-FTD**

Eosu Kim, MD, PhD^1,2,9^, Matthew A. White, PhD^3,9^, Benjamin U. Phillips, PhD^2,4^,

Laura Lopez-Cruz, PhD^2^, Hyunjeong Kim, PhD^1,2^, Christopher J. Heath, PhD^5^,

Jong Eun Lee, PhD^6^, Lisa M. Saksida, PhD^2,7,8^, Jemeen Sreedharan, MRCP, PhD^3*^,

Timothy J. Bussey, PhD^2,7,8*^

^1^Department of Psychiatry, Institute of Behavioral Science in Medicine, Brain Korea 21 Plus Project for Medical Sciences, Yonsei University College of Medicine, Seoul, Republic of Korea.

^2^Department of Psychology and MRC/Wellcome Trust Behavioural and Clinical Neuroscience Institute, University of Cambridge, Cambridge, UK.

^3^Department of Basic and Clinical Neuroscience, Maurice Wohl Clinical Neuroscience Institute, Institute of Psychiatry, Psychology and Neuroscience, King’s College London, London, UK

^4^Department of Physiology, Development and Neuroscience, University of Cambridge, Cambridge, UK.

^5^School of Life, Health and Chemical Sciences, The Open University, Walton Hall, Milton Keynes, UK.

^6^Department of Anatomy, Yonsei University College of Medicine, Seoul, Republic of Korea.

^7^Molecular Medicine Research Laboratories, Robarts Research Institute & Department of Physiology and Pharmacology, Schulich School of Medicine & Dentistry, Western University, London, ON, Canada.

^8^The Brain and Mind Institute, Western University, London, ON, Canada.

^9^These authors contributed equally.

****Correspondence to***

Dr. Jemeen Sreedharan

Department of Basic and Clinical Neuroscience

Maurice Wohl Clinical Neuroscience Institute

King's College London

Institute of Psychiatry, Psychology & Neuroscience

5 Cutcombe Road

London SE5 9RX UK

Email: [jemeen.sreedharan@kcl.ac.uk](mailto:jemeen.sreedharan@kcl.ac.uk)

Dr. Timothy J. Bussey

Western University

Robarts Research Institute

1151 Richmond St N
London, ON

N6A 5B7 Canada

Email: [tbussey@uwo.ca](mailto:tbussey@uwo.ca)


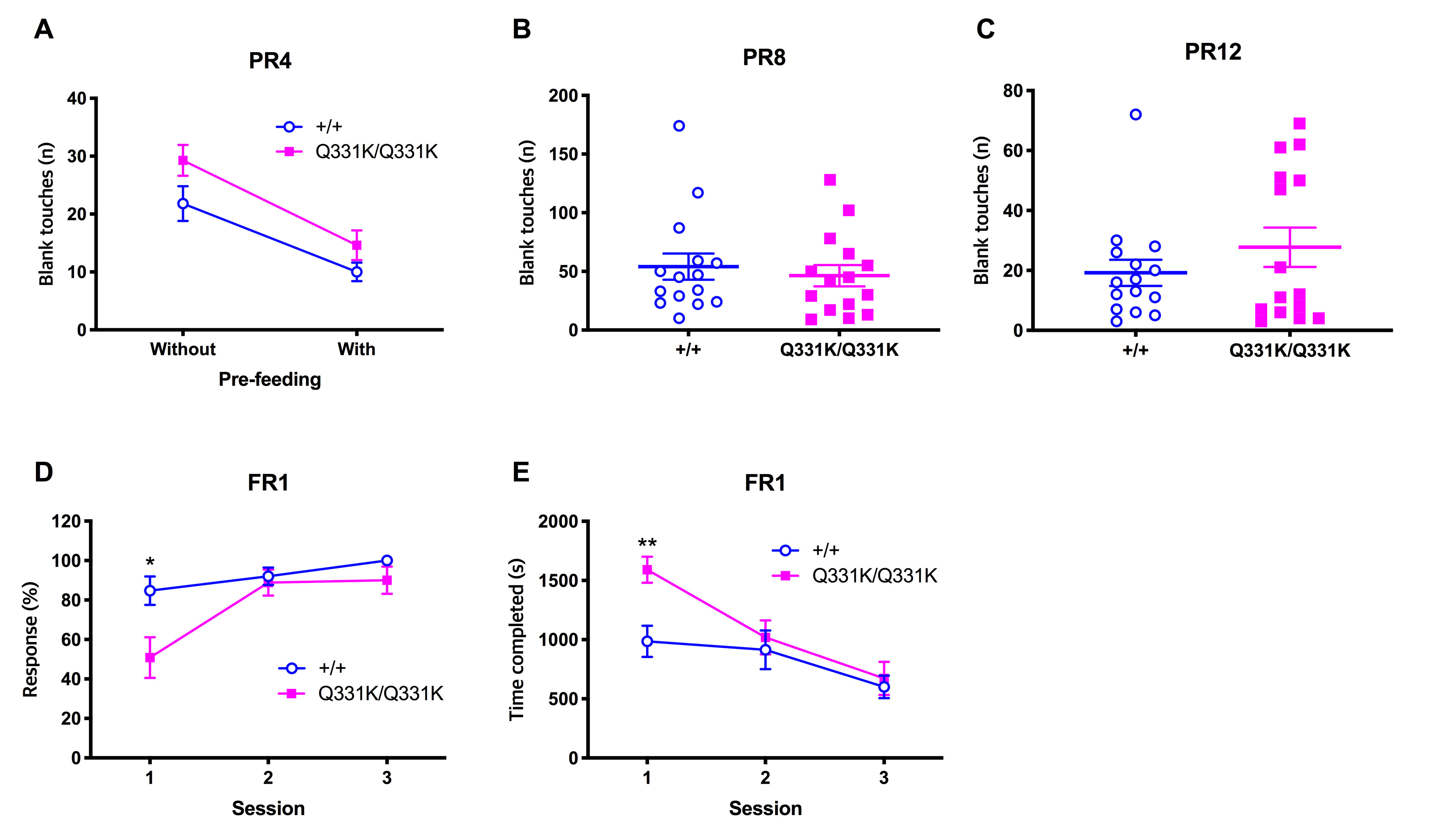


## **Supplementary Figure 1. Blank touches in progressive ratio (PR) schedules and fixed-ratio (FR) schedule acquisition**

(A) The number of blank touches in PR4 with and without prefeeding. Main effect of group, F(1,30) = 1.51, p = 0.228; main effect of prefeeding, F(1,27) = 24.61, p < 0.0001; group x prefeeding interaction, F(1,27) = 0.01, p = 0.913. (B) The number of blank touches in PR8. p = 0.593. (C) The number of blank touches in PR12. p = 0.593. (D) Response % during 30-trial FR1 acquisition. Main effect of group, F(1,30) = 5.19, p = 0.030; main effect of session, F(2,60) = 11.11, p = 0.0001; group x session interaction, F(2,60) = 3.16, p = 0.049; Simple main effect of group, p = 0.018. (E) Time required to complete FR1 session. Main effect of group, F(1,30) = 2.45, p = 0.128; main effect of session, F(2,60) = 19.20, p < 0.0001; group x session interaction, F(2,60) = 3.81, p = 0.027; Simple main effect of group, p = 0.002.


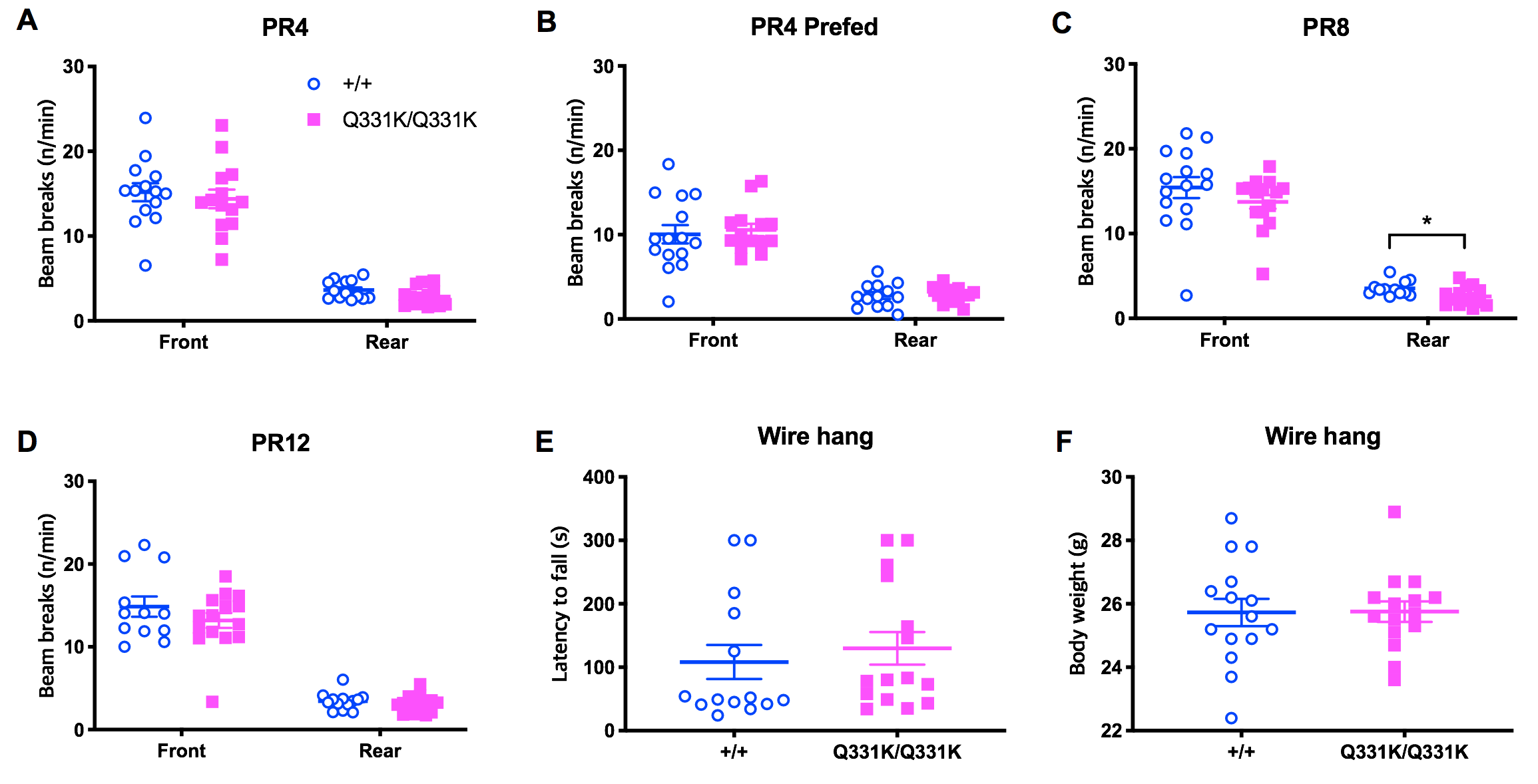


## **Supplementary Figure 2. Locomotor behaviour and muscle strength**

Beam break rates in PR schedules (A-D), which were measured by Infrared bream detectors in the operant chambers. Latency to fall off (E) and body weights (F) in the rolling wire hang test which was capped by 5 min. All p’s = n.s. except the rear beam break rates in PR8 (*p = 0.048). N = 15-16 per genotype.

**Supplementary Figure 3. Visual discrimination**

(A) The number of sessions required to reach criterion (accuracy > 80%), p = 0.005. (B) Response latency toward correct stimulus. Main effect of genotype, F(1,27) = 8.42, p = 0.007. (C) Response latency toward incorrect stimulus. Main effect of genotype, F(1,27) = 8.73, p = 0.006. (D) Latency to collect reward (milkshake) from the magazine. Main effect of genotype, p = n.s. (E) Rate of beam breaks in the front (near touchscreen) side of the operant chamber. Main effect of group; p = 0.316. (F) Rate of beam breaks in the rear (near reward magazine) side. p = 0.330. (G) Mean time staying in the food tray in the magazine for reward consuming. Main effect of group; p = 0.080, group by session interaction; p = 0.035. N = 15 TDP-43^Q331K/Q331K^ and 13 TDP-43^+/+^ mice.

**Supplementary Figure 4. Reversal learning**

(A) The number of sessions required to reach criterion (accuracy > 50%) in reversal learning. p = 0.001. (B) Response latency towards correct stimulus. Main effect of genotype, F(1,24) = 3.60, p = 0.070. (C) Response latency towards incorrect stimulus. Main effect of genotype, F(1,24) = 1.66, p = 0.209. (D) Latency to collect reward (milkshake) from the magazine. Main effect of genotype, p = n.s. (E) Front beam break rate. Main effect of genotype; p = 0.870. (F) Rear beam break rate. p = 0.428. (G) Reward consuming time. p = 0.399. N = 14 TDP-43^Q331K^ and 12 TDP-43^+/+^ mice.


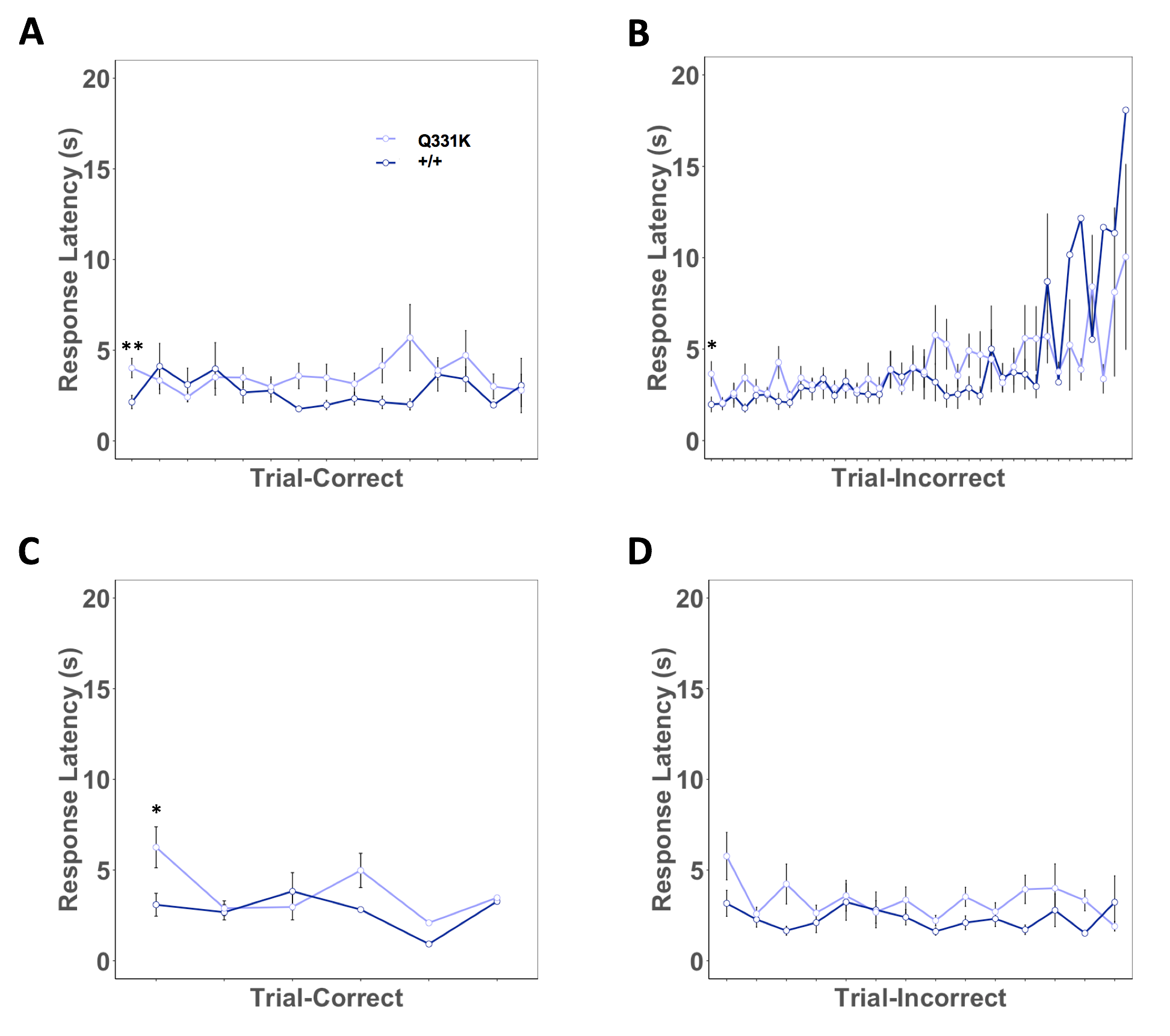


**Supplementary Figure 5:** Slow responding of mutant mice on the first trial in the first sessions of visual discrimination and reversal learning. Response latencies across trials in the first session of pairwise visual discrimination (A, B) and reversal (C,D) learning. (A) Correct response latency; main effect of group, p = 0.076; main effect of trial; p = 0.516; group by trial interaction, p = 0.117. (B) Incorrect response latency; main effect of group, p = 0.080; main effect of trial; p < 0.001; group by trial interaction, p = 0.022. (C) Correct response latency; main effect of group, p = 0.121; main effect of trial; p = 0.103; group by trial interaction, p = 0.168. (D) Incorrect response latency; main effect of group, p = 0.106; main effect of trial; p = 0.006; group by trial interaction, p = 0.307. Simple main effects of group were indicated only for each first trial; *p < 0.05, ** p < 0.01. (A, B) N = 15 TDP-43^Q331K/Q331K^ and 13 TDP-43^+/+^ mice. (C,D) N = 14 TDP-43^Q331K^ and 12 TDP-43^+/+^ mice.
